# The Influence of Mindfulness-Enhanced Resistance Training Program on the Subjective Well-Being of Female College Students: A Randomized Controlled Trial

**DOI:** 10.3390/bs16040553

**Published:** 2026-04-08

**Authors:** Ping Qu, Fang-Bin Li, Yi-Wen Zhou, Feng Pan

**Affiliations:** 1 Department of Physical Education, Sun Yat-sen University, Guangzhou 510275, China; quping@mail.sysu.edu.cn (P.Q.); lifb@mail2.sysu.edu.cn (F.-B.L.); 2 Department of Physical Education, Shenzhen Polytechnic University, Shenzhen 518055, China

**Keywords:** mindfulness, resistance training, self-esteem, subjective well-being, mediating effect, female college students

## Abstract

This study evaluates the effects of a 30-week mindfulness-enhanced resistance training (MRT) program on the physical and mental health of female college students and explores whether changes in self-esteem or mindfulness mediate the relationship between MRT and subjective well-being. Sixty-four healthy female college students were randomly assigned to either the MRT or resistance training (RT) group. Both groups participated in 90 min weekly sessions for 30 weeks. A 2 × 2 mixed-design ANOVA analyzed the intervention’s effects on physical health, mindfulness, self-esteem, and subjective well-being. PROCESS macro (Model 4) tested mediation effects. MRT and RT significantly improved physical health, with MRT showing superior improvements in waist-to-hip ratio, flexibility, and vital capacity. Only MRT improved mindfulness, self-esteem, and subjective well-being. Self-esteem changes fully mediated the relationship between MRT and subjective well-being. MRT as a comprehensive mind–body intervention significantly enhanced the physical health and subjective well-being of female college students, outperforming resistance training. Improvements in self-esteem mediated the relationship between MRT and increased subjective well-being. MRT can serve as an effective approach to promote the physical and mental health of female college students.

## 1. Introduction

In recent years, physical and mental health issues among college students have become increasingly prominent worldwide (Paiva et al., 2025; Shaaban et al., 2026). In China, female college students are particularly affected due to low levels of physical activity and prolonged sedentary behavior (M. M. Guo et al., 2022). Research indicates that their physical fitness and cardiorespiratory endurance have been steadily declining (L. Guo et al., 2025), while the prevalence of mental health issues has remained consistently high (Gao et al., 2020; Lin et al., 2025). Meanwhile, mental health research has gradually shifted from focusing solely on “negative symptoms” such as depression and anxiety to emphasizing the assessment of “positive mental health,” centered on subjective well-being, including life satisfaction and positive emotions (Bohlmeijer & Westerhof, 2021; Magalhães, 2024). Subjective well-being is not only a key dimension of positive mental health (Keyes, 2002), but also a crucial indicator closely related to health outcomes (Steptoe et al., 2015). Compared to their male counterparts, female college students in China exhibit more pronounced emotional distress and a greater decline in subjective well-being (Cheng et al., 2025; Zhu et al., 2025). Therefore, how to improve physical health and subjective well-being in female college students through scalable and sustainable lifestyle interventions in the university setting has become a key issue in the fields of sport psychology and health promotion.

Resistance training (RT), as a key exercise modality for improving muscle strength and mass, has gained increasing attention in both clinical and public health fields in recent years. Systematic evidence shows that structured resistance training is safe and effective for adults, significantly improving physical fitness related to physical health (Ramos-Campo et al., 2021); it can also have a positive impact on mental health (Banyard et al., 2025). In young populations, resistance training helps improve physical fitness, body image, and body-esteem, which may, in turn, indirectly enhance subjective well-being through more positive self-evaluations and greater body satisfaction (White et al., 2024).

However, existing evidence on psychological outcomes remains unclear. Several studies focusing on young adults and adolescents have shown that resistance training alone does not lead to significant improvements in positive psychological outcomes, such as self-esteem and subjective well-being (Smith et al., 2018). Furthermore, compared to a resting state (i.e., non-active control condition), this type of training did not show a clear advantage in improving depressive mood (O’Sullivan et al., 2025). Several factors may account for this finding. In resistance training, the intensity and physical load are often associated with negative experiences such as fatigue and muscle discomfort, which may lead to a decrease in immediate pleasure and subsequently affect psychological benefits (Faro et al., 2019). Additionally, lower levels of autonomous motivation and perceived sense of control may limit the long-term psychological benefits of resistance training (Martinez Kercher et al., 2024). In the absence of strategies that promote self-awareness and emotional experiences, these negative feelings and motivational barriers may further hinder the sustained improvement of psychological benefits (Ten Hoor et al., 2025). In light of these limitations, researchers have begun to explore complementary psychological strategies that may enhance the mental health benefits of resistance training.

Mindfulness interventions, characterized by present-moment awareness and non-judgmental acceptance, have been widely applied in the field of mental health management. A large body of cross-sectional and longitudinal studies has shown a positive correlation between mindfulness training and subjective well-being (Karakurt & Durmaz, 2025; Ma & Xiang, 2023; Michaelsen et al., 2023). Mindfulness practice is also associated with lower emotional reactivity, reduced perceived stress, and fewer maladaptive behavioral patterns (Keng et al., 2011). Furthermore, evidence indicates that mindfulness-based interventions can alleviate negative emotional states, such as anxiety and depression, in various populations (Hofmann et al., 2010). Based on this, traditional mindfulness-based exercises, such as Tai Chi and yoga, which inherently incorporate mindfulness elements (Jankauskiene & Baceviciene, 2024; A. C. Lee et al., 2017), have been widely applied in the field of health promotion. Previous studies have suggested that these practices can improve stress levels (Qi et al., 2022) and anxiety and depression in college students (H. Li et al., 2023), thereby facilitating a comprehensive improvement in mental health. However, existing evidence also indicates that the beneficial effects of these traditional practices on mental health are not always consistent, particularly in relation to positive psychological indicators such as mindfulness levels and subjective well-being (Dawson et al., 2020). Meanwhile, a recent meta-analysis focusing on college students found that the effects of mindfulness interventions alone on emotional symptoms exhibit a certain degree of heterogeneity (Alrashdi et al., 2024). The improvement in positive psychological indicators, such as subjective well-being, is not stable (Dawson et al., 2020; Gong et al., 2023), and there are significant individual differences in the benefits. In the university context, single-session mindfulness courses face practical challenges, such as limited student engagement, high resource investment (Huberty et al., 2019) and insufficient adherence (Canby et al., 2021; Z. Zhang et al., 2024). These issues underscore the necessity of exploring “mind–body integration” strategies.

To enhance the stability of intervention effects and the clarity of underlying mechanisms, recent studies have innovatively attempted to integrate mindfulness training with traditional mindfulness-based exercises (such as yoga and Tai Chi). This integration involves embedding strategies like breath awareness, body scanning, and mindfulness cues during practice, resulting in more consistent or more efficient physical and mental benefits (Qu et al., 2024; J. Y. Zhang et al., 2018). Meanwhile, exercise modalities that integrate mindfulness training have expanded beyond traditional mindfulness-based practices to include a broader range of physical activities, particularly aerobic exercises characterized by repetitive, cyclical movements, such as mindful walking, mindful running, and other non-traditional forms of mindfulness-integrated exercise (Rotter et al., 2022; Zieff et al., 2024). By incorporating attention anchors and present-moment awareness training into these exercises, practitioners are encouraged to maintain a mindful state continuously. These studies also suggest that such practices have potential value in improving physical and mental health (Díaz-Silveira et al., 2025; Remskar et al., 2024). The studies described above are summarized in Table 1.

The studies on mindfulness-integrated exercise mentioned above have made significant contributions to the theory and practice of positive psychology. However, despite these promising findings, the applicability of existing mindfulness-integrated exercise trials to college populations remains limited for several reasons. The exercise modalities used in existing studies, such as yoga, are generally preferred by middle-aged women (Cramer et al., 2016), while Tai Chi, Qigong, and walking are more commonly practiced by middle-aged and older adults (Vergeer et al., 2017). This suggests that current research may need to integrate mindfulness with exercise forms that are more readily accepted by college students and offer significant physiological benefits, in order to provide more effective mind–body promotion strategies for this population.

As one such option, RT is widely practiced and well regarded among college students, producing relatively rapid and noticeable gains in strength and body morphology that can boost self-efficacy and body esteem (Collins et al., 2019). Incorporating mindfulness into this context may further amplify these benefits: by encouraging individuals to cultivate open, nonjudgmental awareness of their breath, muscular effort, and fatigue, mindfulness helps reduce tension and negative physical sensations during exercise, thereby enhancing enjoyment and fostering a greater sense of purpose in the workout experience (Cox et al., 2018). Additionally, mindfulness practice contributes to the enhancement of body image and self-efficacy during physical activity, further optimizing the exercise experience (Jankauskiene & Baceviciene, 2024; L. Li et al., 2023). The positive psychological benefits associated with both interventions can exert a positive impact on subjective well-being (C.-L. Lee et al., 2025; Song et al., 2025). Building on these psychological connections, prior research also suggests that self-esteem may serve as a psychological pathway linking physical activity and mindfulness to subjective well-being (J. Lee et al., 2023; Shang et al., 2021), warranting further investigation as a potential mediator in combined mind–body interventions.

Based on the above discussion, existing integrated intervention strategies primarily include aerobic exercises and traditional mindfulness-based training methods involving repetitive cyclical practices. However, these interventions may be influenced by demographic factors such as gender and age. Currently, there is insufficient evidence to support interventions centered around MRT, despite the fact that RT is more readily accepted and practiced by college students. Furthermore, most studies have relatively short durations and lack long-term data on intervention effects, particularly in long-term randomized controlled trials (RCTs) focusing on the comprehensive impact and mechanisms of these interventions on the physical and mental health of Chinese female college students, where relevant evidence remains limited. To address this limitation, the present study conducts a long-term RCT in which resistance training serves as the primary exercise modality, integrated with mindfulness training. The study compares MRT to RT, with the aim of evaluating its effectiveness in improving physical health, mindfulness, self-esteem, and subjective well-being. Furthermore, the present study develops a mediation model to test whether changes in self-esteem or mindfulness mediate the observed effects of MRT on subjective well-being. This study aims to provide evidence-based support for a combined intervention model using RT as a core modality, along with its psychological mechanisms, for promoting “mind–body integration” health in female college students.

## 2. Materials and Methods

### 2.1. Participants

A priori power analysis was conducted to determine sample size. Sample size calculation was performed using G*Power 3.1 software (Heinrich-Heine-Universität Düsseldorf, Düsseldorf, Germany), with prior research indicating a medium effect size (range 0.25–0.4). Based on this, a type I error rate (α) of 0.05 was set, and a medium effect size (*d* = 0.25) was parameterized for each outcome variable. The statistical power was set at 0.8, which determined that a minimum of 48 participants was required. Considering potential dropout and attrition during the COVID-19 pandemic, a total of 64 female college students from Sun Yat-sen University who met the inclusion criteria were recruited for this study. Participants were randomly assigned to the mindfulness-enhanced resistance training MRT (*n* = 32) or resistance training RT (*n* = 32) groups. During the intervention, nine participants withdrew due to non-study-related factors impacting adherence, including one from the MRT (experimental group) and eight from the RT group. Throughout the testing process, evaluators remained blinded to group assignments to ensure objective data collection. All participants lived on campus and were instructed to maintain their usual daily routines and eating habits during the intervention to minimize the influence of non-intervention factors on the study outcomes. Table 2 shows the general characteristics of the participants.

This study was approved by the Ethics Committee of the Department of Psychology at Sun Yat-sen University prior to the intervention (approval number: 2021-1105-0213) and prospectively registered with the Chinese Clinical Trial Registry (ChiCTR: registration number: 2200058449). An informed consent form for the mindfulness intervention was prepared in accordance with the study protocol. All participants voluntarily agreed to participate in the study and provided written informed consent.

Inclusion criteria: (1) Female undergraduate students in their first or second year at Sun Yat-sen University; (2) Have no prior experience with structured mindfulness training or organized resistance training; (3) Adhere to a healthy lifestyle, regularly engage in physical activities excluding resistance training, and maintain a balanced diet; (4) In optimal health, with no history of high blood pressure, coronary artery disease, diabetes, physical disabilities, or musculoskeletal disorders; (5) Willing to participate in the study and provide written informed consent.

Withdrawal criteria: (1) Withdrawal from the study due to injury or other unforeseeable circumstances during the intervention period; (2) Poor adherence, defined as completing less than 85% of the prescribed weekly training volume (Müller et al., 2021).

Participants may compensate for missed sessions within the same week; failure to attend at least 85% of the scheduled sessions will result in removal from the study, with such cases classified as sample attrition.

**Figure 1:**
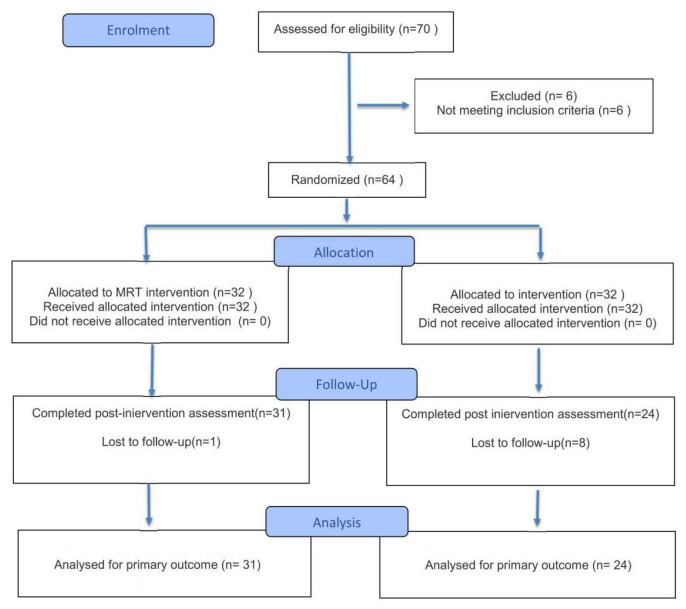
Flow chart of experimental object selection.

The flow of participant screening and allocation is illustrated in Figure 1, and baseline characteristics are presented in Table 2.

### 2.2. Experimental Design and Procedures

(1) MRT (experimental group): The intervention content and format were jointly developed by faculty members from the Department of Physical Education and the Department of Psychology at Sun Yat-sen University. The resistance training instructor had three years of experience in mindfulness practice and instruction and ten years of experience in resistance training and teaching.
(2) RT (control group): The intervention was delivered by an instructor from the Department of Physical Education at Sun Yat-sen University with ten years of experience in resistance training and teaching.

### 2.3. Experimental Intervention Scheme

Both the MRT and RT groups underwent a 30-week intervention, with sessions held once a week for 90 min. The intervention for the RT group followed the curriculum and content requirements of the first-year “Fitness” course in the Department of Physical Education at Sun Yat-sen University, primarily focusing on resistance training exercises. During RT, the instructor did not introduce mindfulness explicitly nor guide participants to approach the resistance exercises with a mindfulness-centered mindset. The resistance training protocol in the MRT group was identical to that of the RT group. However, there were minor differences in the time allocation between the preparatory and main phases of each session. Additionally, the two groups performed their concentrated practice sessions at different class times, as detailed in Table 3. The MRT group followed a training intervention plan developed by the faculty team described in Section 2.2. Each session began with resistance training, mindfulness, and a round of sensory sharing and training content introduction (15 min). The instructor then guided participants through dynamic warm-ups, including cardiovascular, stretching, and core exercises (10 min), followed by a series of resistance training exercises (45 min). Throughout the practice, the instructor provided continuous verbal guidance, encouraging participants to maintain mindfulness. Participants were guided to focus on their breath while non-judgmentally noticing their internal state and muscle sensations, actively embracing challenges. During rest periods, a 10 min group mindfulness practice was conducted, including body scanning, loving-kindness meditation, and breath awareness exercises. The RT group did not engage in any exercises during the rest periods. Following the rest, participants completed 10 min of stretching and relaxation, followed by a session summary. A detailed comparison of the MRT and RT groups in terms of intervention content, teaching methods, designated exercises, and time allocation is provided in Table 3, Table 4 and Table 5 and the Supplementary Materials.

### 2.4. Measures

#### 2.4.1. Psychological Indicators

(1) Self-esteem level

The self-esteem data in this study were measured using the Chinese version of the Rosenberg Self-Esteem Scale, translated by Ji and Yu (1993), from Rosenberg (1965), which consists of 10 items, with 4 items scored reversely. The scale uses a 4-point Likert scale, ranging from 1 (strongly disagree) to 4 (strongly agree). This scale has been validated for use with Chinese college students and has shown acceptable reliability and validity (Peng et al., 2024). In this study, the overall Cronbach’s α coefficients for the pre-test and post-test were 0.804 and 0.782, respectively, meeting the basic requirements for reliability and validity.

(2) Mindfulness level

Mindfulness was measured using the Chinese version of the Five Facet Mindfulness Questionnaire-SF (FFMQ-SF) (Bohlmeijer et al., 2011), translated by Hou et al. (2014). This scale includes 20 items that reflect five dimensions of mindfulness: observing, describing, acting with awareness, non-judging, and non-reactivity. The score range is from 20 to 100, with higher scores indicating higher levels of mindfulness. This scale has been validated for use with Chinese college students and has shown acceptable reliability and validity (Dai et al., 2022). The overall Cronbach’s α coefficients for the pre-test and post-test were 0.808 and 0.804, respectively, meeting the basic requirements for reliability and validity.

(3) Subjective well-being

The subjective well-being data in this study were measured using the Chinese version of the Subjective Happiness Scale (Campbell, 1976), translated by J. Li and Zhao (2000). The scale consists of two components: the overall emotional index and life satisfaction, with a total of nine items, using a 7-point Likert scale. The overall happiness index score is calculated by averaging the scores of the two components (weighted 1:1). Higher scores indicate a higher level of happiness. This scale has been validated for use with Chinese college students and has shown acceptable reliability and validity (Qu et al., 2026). In this study, the Cronbach’s α coefficients for the pre-test and post-test were 0.879 and 0.939, respectively, meeting the basic requirements for reliability and validity.

#### 2.4.2. Physical Health Indicators

**Figure 2:**
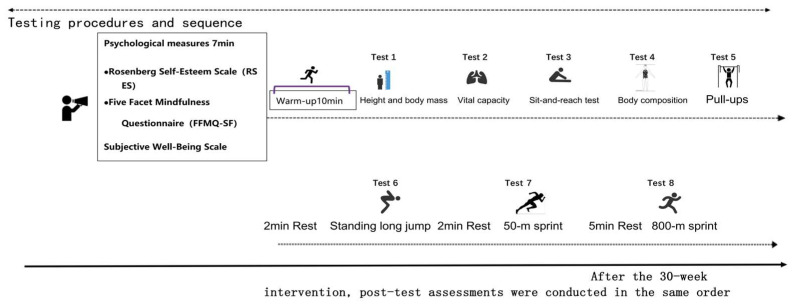
Testing procedures and sequences.

According to the physical health testing standards for female college students based on the Chinese Student Physical Health Standards (Bian, 2017), the physical health level was reflected by the overall score derived from three major categories: body morphology (body fat percentage, waist-to-hip ratio), physical fitness (endurance/800 m run, explosive power/standing long jump, muscular strength/sit-ups, flexibility/sit-and-reach, speed/50 m run), and physical function (vital capacity index). Since body fat percentage in body morphology, as well as the 800 m run and 50 m run in physical fitness, are reverse indicators (i.e., a smaller measurement value indicates better performance), reverse-scored items were transformed to ensure consistency with the direction of other indicators. The final analysis used standardized Z-scores, T-scores, and reverse-transformed scores for these physical health indicators, which were then summed to create a composite score (Figure 2). In the present study, total physical health score refers to the composite score derived from standardized indicators, whereas health-related physical fitness refers to specific physical components associated with health outcomes.

### 2.5. Statistical Methods

SPSS 26.0 statistical software was used to test the differences in the pre-test data and post-test data, and the normal distribution, variance homogeneity and descriptive statistics of the data were tested in turn. A mixed-design analysis of variance (ANOVA) was conducted to test the interaction between group (MRT group/RT group) and time (pre-test/post-test). If the interaction was significant, further simple effects tests for time and group were performed. Post hoc pairwise comparisons were conducted using the Bonferroni correction, and effect sizes were reported using partial η^2^ (0.01: small effect, 0.06: moderate effect, 0.14: large effect) (Cohen, 1977). A *p*-value less than 0.05 was regarded as statistically significant. Simple mediation analysis was conducted using the Process plugin with 5000 bootstrap samples. The significance of the mediation effect was determined by a 95% confidence interval that did not include zero (Preacher & Hayes, 2008).

## 3. Results

### 3.1. Baseline Differences

No significant baseline differences were found between the MRT and RT groups in terms of demographic characteristics or study variables (*p* > 0.05). Descriptive statistics for the study variables of both groups are presented in Table 6.

### 3.2. Interaction Effects (Time × Group)

The results of the mixed-design ANOVA showed a significant interaction between time and group when the total physical health score was used as the dependent variable (*F* [1, 53] = 4.46, *p* = 0.040, η^2^ = 0.078).

The interaction between time and group was significant for waist-to-hip ratio (*F* [1, 53] = 4.31, *p* = 0.043, η^2^ = 0.075), sit-and-reach test (*F* [1, 53] = 7.69, *p* < 0.001, η^2^ = 0.127), and vital capacity (*F* [1, 53] = 6.37, *p* = 0.015, η^2^ = 0.107).

For the psychological outcomes, the interaction effects were similarly significant. The analysis yielded significant Group × Time interactions for mindfulness (*F* [1, 53] = 8.04, *p* = 0.006, η^2^ = 0.132), self-esteem levels (*F* [1, 53] = 7.52, *p* = 0.008, η^2^=0.124), and subjective well-being (*F* [1, 53] = 5.54, *p* = 0.022, η^2^ = 0.095).

### 3.3. Within-Group Simple Effects

Simple effects analysis within groups indicated that both MRT (*MD* = 53.50, *F* [1, 53] = 193.65, *p* < 0.001, η^2^ = 0.785) and RT (*MD* = 41.21, *F* [1, 53] = 88.97, *p* < 0.001, η^2^ = 0.627) showed significant increases in physical health scores post-intervention.

For the MRT group, waist-to-hip ratio (*MD* = −0.02, *F* [1, 53] = 15.68, *p* < 0.001, η^2^ = 0.228), sit-and-reach test (*MD* = 4.78, *F* [1, 53] = 50.64, *p* < 0.001, η^2^ = 0.489), and vital capacity (*MD* = 607.52, *F* [1, 53] = 87.78, *p* < 0.001, η^2^ = 0.613) all showed significant improvements post-intervention.

In the RT group, significant improvements were also found for the sit-and-reach test (*MD* = 1.96, *F* [1, 53] = 6.59, *p* = 0.013, η^2^ = 0.111) and vital capacity (*MD* = 354.00, *F* [1, 53] = 22.02, *p < 0.001*, η^2^ = 0.294). However, the change in waist-to-hip ratio for the RT group was not significant (*MD* = 0, *F* [1, 53] = 0.519, *p* = 0.474, η^2^ = 0.010).

For psychological indicators, the MRT group showed significant increases in mindfulness (*MD* = 5.97, *F* [1, 53] = 21.62, *p* < 0.001, η^2^ = 0.290), self-esteem levels (*MD* = 2.28 *F* [1, 53] = 4.82, *p* = 0.033, η^2^ = 0.083), and subjective well-being (*MD* = 4.12, *F* [1, 53] = 6.83, *p* = 0.012, η^2^ = 0.114) following the intervention. However, no significant changes were observed in these psychological variables in the RT group (*p* > 0.05).

### 3.4. Main Effects

The main effect of group was not statistically significant for body fat percentage (*F* [1, 53] = 2.22, *p* = 0.142, η^2^ = 0.040), 800 m run time (*F* [1, 53] = 1.58, *p* = 0.214, η^2^ = 0.029), standing long jump distance (*F* [1, 53] = 0.11, *p* = 0.745, η^2^ = 0.002), 50 m sprint time (*F* [1, 53] = 0.38, *p* = 0.541, η^2^ = 0.007), and sit-up count (*F* [1, 53] = 1.13, *p* = 0.292, η^2^ = 0.021; Table 7).

The main effect of time was significant: body fat percentage (*F* [1, 53] = 45.03, *p* < 0.001, η^2^ = 0.459), 800 m run time (*F* [1, 53] = 13.628, *p* = 0.001, η^2^ = 0.205), standing long jump distance (*F* [1, 53] = 32.16, *p* < 0.001, η^2^ = 0.378), 50 m sprint time (*F* [1, 53] = 54.52, *p* < 0.001, η^2^ = 0.507), and sit-up count (*F* [1, 53] = 215.07, *p* < 0.001, η^2^ = 0.802).

**Figure 3:**
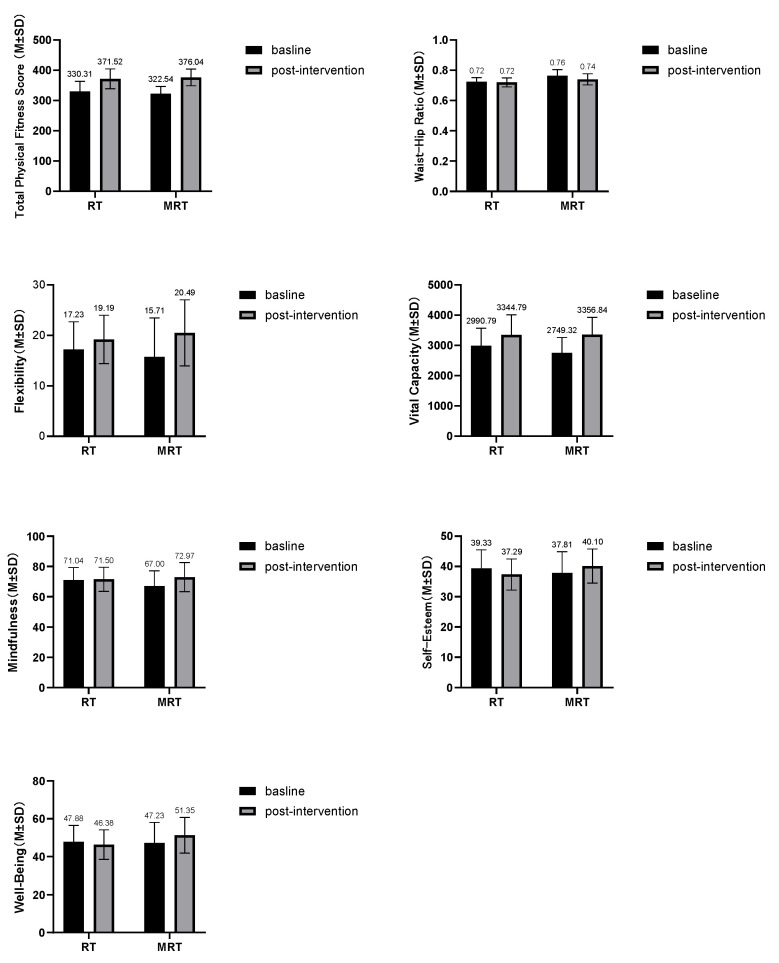
Changes in the mean values of physical and psychological indicators of MRT and RT subjects before and after intervention. Note: (1) MRT refers to the Mindfulness-Enhanced Resistance Training Group, and RT refers to the Resistance Training Group; (2) *“M”* denotes the mean. *“SD”* denotes standard deviation.

Post hoc comparisons confirmed that both groups improved significantly from pre-test to post-test on these measures: body fat percentage (MRT *MD* = −4.58, RT *MD* = −3.25), 800 m run time (MRT *MD* = −13.71, RT *MD* = −7.33), standing long jump distance (MRT *MD* = 8.13, RT *MD* = 6.83), 50 m sprint time (MRT *MD* = −0.44, RT *MD* = −0.55), and sit-up count (MRT *MD* = 12.68, RT *MD* = 13.37). However, consistent with the non-significant interaction effects, the magnitude of these improvements did not differ significantly between the two groups (*p* > 0.05; Table 7, Figure 3).

### 3.5. Mediation

**Figure 4:**
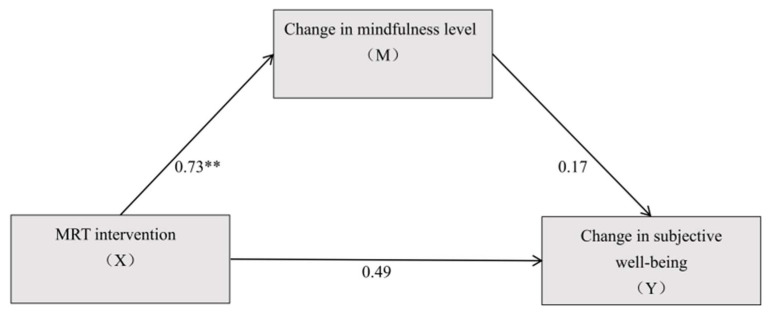
The mediating effect of mindfulness. Note: ** *p* < 0.01.

Group was treated as the independent variable (MRT = 1, RT = 0) and dummy-coded for the mediation analysis, with mindfulness level as the mediator and changes in subjective well-being as the dependent variable for the mediation analysis. The results (Figure 4, Table 8 and Table 9) showed that group significantly predicted changes in subjective well-being (β = 5.629, *t* = 2.354, *p* < 0.05). Additionally, group significantly predicted mindfulness levels (β =5.509, *t* = 2.835, *p* < 0.01). When both group and changes in mindfulness were included, group (β = 4.480, *t* = 1.754, *p* > 0.05) did not significantly predict subjective well-being, and mindfulness level did not significantly predict subjective well-being either (β = 0.209, *t* = 1.240, *p* > 0.05).

Further examination of the mediation effect revealed that, after including changes in mindfulness level, the 95% confidence interval for the direct effect of group on changes in subjective well-being included zero, with a Bootstrap (95% CI = [−0.6450, 9.6047]). Similarly, the 95% confidence interval for the mediation effect of changes in mindfulness level also included zero, with a Bootstrap (95% CI = [−1.0895, 4.9155]), indicating that the mediation effect of changes in mindfulness level was not significant. The direct effect (4.480) and the mediation effect (1.149) accounted for 79.59% and 20.41% of the total effect (5.629), respectively.

**Figure 5:**
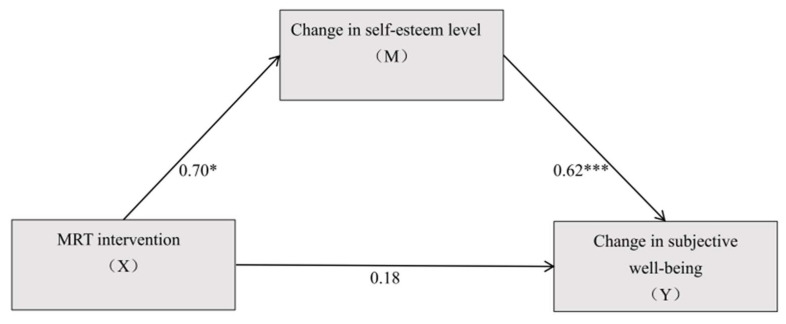
The mediating effect of self-esteem. Note: * *p* < 0.05, *** *p* < 0.001.

Group was treated as the independent variable (MRT = 1, RT = 0) and dummy-coded for the mediation analysis, with changes in self-esteem as the mediator and changes in subjective well-being as the dependent variable for the mediation analysis. The results (Figure 5, Table 8 and Table 9) showed that group significantly predicted changes in subjective well-being (β = 5.629, *t* = 2.354, *p* < 0.05). Additionally, group significantly predicted changes in self-esteem (β = 4.332, *t* = 2.742, *p* < 0.01). When both group and changes in self-esteem were included, group (β = 1.614, *t* = 0.791, *p* > 0.05) no longer significantly predicted the dependent variable, while changes in self-esteem still significantly predicted changes in subjective well-being (β = 0.927, *t* = 5.582, *p* < 0.001).

Further examination of the mediation effect revealed that, after including changes in self-esteem, the direct effect of group on changes in subjective well-being had a 95% confidence interval that included zero, with a Bootstrap (95% CI = [−2.4801, 5.7085]). In contrast, the 95% confidence interval for the mediation effect of changes in self-esteem did not include zero, with a Bootstrap (95% CI = [0.9607, 8.1125]), indicating a significant mediation effect of self-esteem changes, which was a full mediation. The direct effect (1.614) and the mediation effect (4.015) accounted for 28.68% and 71.32% of the total effect (5.629), respectively.

## 4. Discussion

This study compared the effects of a 30-week MRT and RT on the physical and mental health of female college students. It also investigated whether changes in self-esteem and mindfulness mediated the relationship between intervention type and changes in subjective well-being within the MRT model. Results indicated that both MRT and RT significantly improved physical health outcomes (including body morphology, physical fitness, and physical function) among participants. However, MRT was more effective than RT at improving the waist-to-hip ratio, flexibility, and vital capacity. Only the MRT group showed significant gains in mindfulness, subjective well-being, and self-esteem; no significant changes were observed in these psychological outcomes for the RT group. Mediation analyses revealed that changes in self-esteem fully mediated the effect of intervention type on changes in subjective well-being, whereas changes in mindfulness did not serve as a significant mediator.

Both MRT and RT produced significant improvements across multiple indices of health-related physical fitness, indicating that structured resistance training alone confers substantial health-promoting benefits. This finding is consistent with prior evidence demonstrating the positive effects of resistance training on physical fitness outcomes (E. D. Lee et al., 2022; Ramos-Campo et al., 2021). The additional improvements in flexibility and vital capacity observed in the MRT condition may be attributed to the integration of mindful breathing, an attitude of acceptance, and guided body awareness during training. Previous research indicates that performing resistance training through a full range of motion at the joints can enhance flexibility to a degree comparable to that achieved with static stretching—while simultaneously increasing muscular strength (Alizadeh et al., 2023). In the present study, participants in the MRT group may have been more willing to embrace the benefits of resistance and stretching exercises with a mindful stance, engage more fully in practice, tolerate training-related discomfort with greater self-compassion, and monitor movement amplitude more precisely, thereby promoting more standardized execution of both exercise and stretching and ultimately producing greater improvements in flexibility than RT (Wang et al., 2021). In addition, integrating breathing exercises with resistance training, or combining slow diaphragmatic breathing with strength exercise, may further enhance respiratory muscle function (Fan et al., 2024). In the MRT protocol implemented in the present study, motor learning emphasized breath–movement coordination, maintenance of a neutral spine, and mindful awareness of antagonist muscle relaxation. Over the 30-week intervention, these practice elements may have enhanced trunk stability and thoracic mobility, thereby contributing to an apparent additive benefit in both flexibility and vital capacity. Notably, despite a shorter duration of active exercise, the MRT group achieved health-related fitness improvements comparable to those observed in the RT group and even demonstrated superior gains in selected outcomes, underscoring the potential advantages and practical value of this integrated mind–body intervention.

Beyond the physical adaptations observed, the present findings further highlight distinct psychological mechanisms underlying the MRT intervention. The present study demonstrated that MRT significantly enhanced participants’ mindfulness, self-esteem, and subjective well-being, consistent with prior findings. By encouraging participants to engage in training with a mindful stance and to cultivate mindfulness-related habits and dispositions, the intervention may have strengthened momentary mindfulness experiences that subsequently generalized to daily life, thereby increasing trait mindfulness (Kiken et al., 2015). In parallel, mindfulness practice may facilitate more adaptive self-referential processing (Saraff et al., 2020) and attenuate negative self-directed affect; together, these changes in self-appraisals may ultimately contribute to higher self-esteem (Goldin & Gross, 2010). Evidence from college samples further suggests that physical activity can indirectly improve mental health through increases in mindfulness and self-esteem, with a significant serial mediation pathway in which physical activity influences psychological outcomes via mindfulness and then self-esteem, implying that mindfulness may bolster self-esteem and thereby promote more favorable psychological functioning (Tian & Yang, 2024). Moreover, mindfulness has been positively associated with self-esteem, and specific mindfulness facets (e.g., awareness and non-judgment) have been shown to predict variance in self-esteem, supporting a mechanistic account whereby mindfulness training may facilitate improvements in self-esteem (Chandna et al., 2022). From the perspective of self-determination theory, when individuals experience autonomy, competence, and relatedness through engagement in behavior, satisfaction of these basic psychological needs can strengthen self-worth and self-esteem, thereby enhancing intrinsic motivation and fostering positive affective change (Ryan & Deci, 2000). MRT may enhance perceived competence by directing participants’ attention to bodily changes and incremental progress, thereby strengthening their appraisals of personal capability. Self-compassionate language used during sessions may further encourage participants to relate to their bodies in a nonjudgmental manner, supporting autonomy by enabling them to experience, interpret, and evaluate bodily states more freely. In addition, the group-based format may cultivate interpersonal support and a sense of belonging. Together, these elements may facilitate increases in self-esteem by promoting more positive self-evaluations. Prior findings regarding the effects of RT on self-esteem have been mixed (Moore et al., 2011; Smith et al., 2018). In the present study, self-esteem in the RT group did not improve significantly after the prolonged training period. This null effect may be attributable, at least in part, to the relatively high baseline level of self-esteem in the RT sample (i.e., upper–moderate range), which could have limited observable gains (Marsh et al., 2010; Smith et al., 2018).

The present study demonstrated that changes in self-esteem fully mediated the association between MRT and subjective well-being. This mechanism is consistent with accumulating evidence indicating that self-esteem represents a central psychological resource linking physical activity to subjective well-being, with effects largely transmitted through indirect pathways rather than a robust direct effect (Liao et al., 2023; Pan et al., 2025; Yao et al., 2025). Individuals with higher self-esteem tend to cope more effectively with negative affect and regulate emotional fluctuations more adaptively, which supports more stable positive affect and higher life satisfaction (Kraiss et al., 2020; Sanchez-Sanchez et al., 2025). Moreover, increases in self-esteem are closely related to greater self-acceptance, which has been associated with enhanced subjective well-being (Klussman et al., 2022; Szentagotai-Tatar & David, 2013).

From the perspective of self-determination theory, when structured physical activity satisfies basic psychological needs—autonomy, competence, and relatedness—it can strengthen self-worth and foster well-being (Ryan & Deci, 2000). In the MRT context, mindfulness-related cues and self-compassionate guidance may have facilitated participants’ detection and affirmation of positive bodily signals and incremental progress, thereby enhancing positive self-appraisals and self-esteem; in turn, improved self-esteem may contribute to subjective well-being by promoting more adaptive social functioning and greater perceived support (Marshall et al., 2014; Siedlecki et al., 2014; Yuan et al., 2023). Beyond interpersonal pathways, self-esteem enhancement may also bolster self-efficacy and proactive engagement in daily life, which are typically associated with higher subjective well-being (Cattelino et al., 2023; Joshanloo, 2022).

Collectively, these findings support the proposed pathway whereby MRT contributes to subjective well-being through self-esteem enhancement, extending evidence for the “physical activity–self-esteem–subjective well-being” mechanism within a mindfulness-integrated resistance training context (Liao et al., 2023; Pan et al., 2025; Remskar et al., 2024).

Although mindfulness increased significantly in the MRT group, its change did not mediate the association between intervention condition and subjective well-being. Systematic reviews focusing on university students have shown that mindfulness-based interventions typically yield small-to-moderate effects on stress, anxiety, and depression, as well as on subjective well-being (Alrashdi et al., 2024). However, effects on positive psychological outcomes appear comparatively less consistent, with substantial heterogeneity across studies (Gong et al., 2023). Recent reviews further indicate that combining physical activity with mindfulness is generally beneficial for psychological health, yet the existing integrated programs are often characterized by small samples, short intervention periods, and considerable variability in intervention components and delivery (Remskar et al., 2024; Xu et al., 2025). In addition, longitudinal evidence suggests a dose–response relationship for mindfulness practice, such that higher practice frequency, longer session duration, and greater overall training exposure are associated with larger increases in trait mindfulness and more robust psychological benefits (Bowles & Van Dam, 2025; Strohmaier, 2020; Strohmaier & Goldberg, 2024). In the present study, the mindfulness component involved only one structured practice session per week, and participants’ baseline levels of subjective well-being and self-esteem were already in the upper-moderate range. These factors may have jointly constrained the magnitude and variability of mindfulness change, thereby reducing the detectable indirect effect and resulting in a nonsignificant mediation pathway.

This study has several limitations. First, the sample comprised only female university students, and differential attrition between groups (MRT: 3.1%; RT: 25.0%) represents a potential source of bias. Although baseline characteristics did not differ significantly between completers and non-completers, and withdrawals were primarily attributed to non-study-related factors during the COVID-19 period; a formal intention-to-treat analysis was not conducted due to the absence of post-intervention data for withdrawn participants. The per-protocol approach adopted in the present study may have introduced selection bias and could have inflated the observed MRT effects. Second, no post-intervention follow-up was conducted, precluding evaluation of the durability of the effects of resistance training combined with mindfulness components. Third, as an initial investigation of MRT, the outcome set focused on core dimensions closely related to the intervention and participants’ psychosocial functioning (e.g., self-esteem, mindfulness, and subjective well-being). Negative affective outcomes such as anxiety and depression were not assessed, which may limit the comprehensiveness of the psychological evaluation. Fourth, because the trial was conducted during the COVID-19 period, pandemic-related anxiety and stress may have acted as potential confounders influencing the observed effects. Finally, the duration of active exercise was not equivalent between the MRT and RT conditions, which may have affected the comparability of outcome changes across groups. Furthermore, the MRT intervention incorporated additional components beyond mindfulness training per se, including structured group sharing and instructor-guided attention, which may have introduced non-specific psychosocial effects such as social bonding and increased attention. The absence of an active psychological control condition therefore limits the present study’s ability to attribute the observed psychological benefits specifically to the mindfulness component. Notwithstanding these limitations, the present findings offer meaningful insights that can inform the design of future research.

Future studies should recruit larger and more diverse samples including males and other populations to enhance generalizability, incorporate long-term follow-up assessments to evaluate the durability of intervention effects, and broaden the range of outcome domains and measurement instruments to more comprehensively evaluate the physical and psychological benefits of MRT. Additionally, intention-to-treat analysis with appropriate imputation methods should be employed to strengthen internal validity, and an active psychological control condition should be considered to better isolate the specific effects of the mindfulness component.

## 5. Conclusions

The results of this study suggest that the 30-week MRT and RT interventions effectively improve the physical health of female college students. Compared to RT, MRT exhibited significantly greater improvements in enhancing subjective well-being, mindfulness, self-esteem, and several physical indicators, specifically the waist-to-hip ratio, flexibility, and vital capacity. Furthermore, changes in self-esteem fully mediated the relationship between the intervention type and enhancements in subjective well-being. These results suggest that MRT appears to be an efficient and feasible mind–body intervention for promoting holistic health in this population.

## Figures and Tables

**Table 1:** Mindfulness-integrated exercise trials: a comparative summary.

	Intervention Model	Participants	Outcome Measures (Compared to the Control Group)	Limitations of the Study
**Mindfulness + Traditional Mindfulness-Based Exercise**	10 weeks mindfulness-enhanced Tai Chi Chuan (Qu et al., 2024)	119 healthy college students from a comprehensive university in China	Health status and skill-related physical fitness, mindfulness levels ↑, depression ↓, anxiety and stress ↓	The gender differences among participants were not adequately considered.
	8 weeks mindfulness-based Tai Chi Chuan (J. Y. Zhang et al., 2018)	64 college students with subclinical depression from a medical university in China	Mindfulness levels ↑, depression ↓, anxiety and stress ↓	The physical outcome measures are lacking.
	12 weeks Mindfulness-based yoga (Schuver & Lewis, 2016)	40 women with depression from abroad	Depression levels ↓	There are too few psychological outcome measures.
**Mindfulness +** **Non-traditional Mindfulness-Based Exercise**	4 weeks Mindfulness Meditation Combined with Aerobic Exercise (Zieff et al., 2024)	32 university students with high stress from abroad (84% female)	Stress ↓, anxiety and depression ↓	The intervention period was relatively short, and the sample size was small.
	6 weeks Mindfulness Meditation Combined with 60–80% HRmax Aerobic Exercise (Shors et al., 2018)	105 adult women from abroad	Ruminative thinking, self-worth ↑	The physical outcome measures are absent.
	8 weeks mindful walking program (MWP) (Rotter et al., 2022)	55 adult patients with chronic low back pain from abroad	Pain, back function, and perceived stress (no significant effects)	Changes in physical outcome measures were not observed.

Note: ↑ indicates increased or improved outcomes; ↓ indicates decreased or reduced outcomes.

**Table 2:** General information of experimental subjects (*n* = 55).

Group	Number of Participants	Age/Year	Height/cm	Weight/kg
**MRT**	31	18.00 ± 0.58	160.11 ± 5.64	54.72 ± 6.79
**RT**	24	18.04 ± 0.55	162.75 ± 5.06	52.97 ± 5.21

**Table 3:** Comparison table of training arrangements between the two groups.

Group Training Structure	MRT (Experimental Group)		RT (Control Group)	
	Specific Content	Duration	Specific Content	Duration
**Preparation Phase**	Training Introduction + Resistance Training (5 min) Mindfulness and Sensory Sharing (10 min)	15 min	Training Introduction	5 min
	Warm-up: Cardiovascular/Stretching/Core Exercises	10 min	Warm-up: Cardiovascular/Stretching/Core Exercises	20 min
**Main Phase**	Review of Learned Exercises	15 min	Review of Learned Exercises	20 min
	Mindfulness-Enhanced Resistance Training, Group Exercises	30 min	Resistance Training, Group Exercises	35 min
	Mindfulness Group Practice (mainly static) (Body Scanning, Loving-Kindness Meditation, Breath Awareness, etc.)	10 min	Rest	10 min
**Conclusion Phase**	Relaxation + Summary + Independent Tasks	10 min	Relaxation + Summary + Independent Tasks	10 min

**Table 4:** Summary table of intervention comparison between MRT and RT.

	MRT	RT
**Intervention Content**	Set different mindfulness themes; Resistance training exercises targeting the shoulders, chest, hips, legs, arms, and the entire body.	Resistance training exercises targeting the shoulders, chest, hips, legs, arms, and the entire body.
**Teaching Methods**	Guide with a mindfulness attitude, emphasizing breath awareness and increasing awareness of movement posture and positioning.	Emphasize breath awareness and the perception of movement posture and positioning using Resistance training instruction methods.
**Independent Tasks**	Mindfulness storytelling, mindfulness reading materials, mindfulness assignments; Introduction to resistance training diet; Recommendations for resistance training programs; Daily diet and exercise log.	Introduction to resistance training diet; Recommendations for resistance training programs; Daily diet and exercise log.
**Content Distribution for Each Intervention**	Introduction to resistance training, mindfulness sharing; Warm-up; Mindfulness-enhanced resistance training; Mindfulness group practice; Stretching and relaxation, summary, and independent tasks.	Introduction to resistance training; Warm-up; Resistance training; Rest; Stretching and relaxation, summary, and independent tasks.

**Table 5:** Intervention procedures and schedule for MRT.

**Commencement phase (15 min)**	**1. Program Introduction (5 min)** The session commenced with an oral introduction to the weekly mindfulness theme and training content. **2. Mindfulness-based practice and independent tasks group sharing (10 min)**
**Warm up Exercise** **(10 min)**	**Cardiorespiratory Fitness/Stretching/Core Stabilization Exercises (10 min)** Participants were instructed to anchor attention to breathing, redirect cognitive focus to present-moment experience, and cultivate interoceptive awareness of somatic and affective states.
**MRT practice** **(45 min)**	**Perform circuit training consisting of traditional resistance-training exercises targeting the shoulders, chest, hips, legs, arms, and whole-body movements** (Review of previously learned movements, mindfulness-enhanced resistance training, and group practice). Instruct participants to focus on breathing. Begin by anchoring attention to the breath and the present moment. Maintain breath awareness during movement to establish movement rhythm. Conclude by observing the breath and noting one’s state. Instruct participants to observe, without judgment, emergent physical or exercise-related phenomena (including fatigue, soreness, or perceived difficulty) and to accept challenges proactively. Guide participants to recognize correct movement execution and associated bodily sensations to enhance exercise quality and efficiency. Utilize partnered exercises to facilitate the experience of mindful attitudes such as patience, trust, gratitude, and generosity. For participants exhibiting an impatient or overly goal-driven exercise mindset, encourage the application of mindful attitudes such as “non-striving” and “patience”.
**Mindfulness practice** **(10 min)**	Breath Awareness Body Scan Mindfulness Experience Sharing Loving-Kindness Meditation Breathing Space
	Guided mindfulness exercises were administered via live facilitator narration or pre-recorded audio. Participants were given the option to select designated areas and adopt self-selected postures (seated or supine on yoga mats) to optimize physical comfort and relaxation. All participants kept their eyes closed throughout the practice. Participants were invited to verbally articulate somatic and cognitive experiences derived from their previous-week mindfulness practice.
**Final phase** **(10 min)**	Cool-down Stretching Session Debriefing Post-Session Exercise Independent Tasks

**Table 6:** Descriptive statistics and comparison of differences in physical and mental health indicators (*n* = 55).

Indicators	MRT (*n* = 31) (*M* ± *SD*)	RT (*n* = 24) (*M* ± *SD*)	*p*
Height/cm	160.11 ± 5.64	162.75 ± 5.06	0.490
Weight/kg	54.71 ± 6.79	52.98 ± 5.21	0.160
Mindfulness	67.00 ± 10.10	71.04 ± 8.24	0.117
Self-Esteem	37.81 ± 7.04	39.33 ± 6.09	0.587
Subjective Well-Being	47.23 ± 10.86	47.88 ± 8.63	0.811
Total score of physical health	322.54 ± 24.11	330.31 ± 33.27	0.213
Waist-to-hip ratio	0.76 ± 0.04	0.72 ± 0.03	0.064
Vital capacity/mL	2749.32 ± 512.39	2990.79 ± 575.17	0.647
Sit-and-reach test/cm	15.71 ± 7.72	17.23 ± 5.46	0.086
Body fat percentage/%	24.90 ± 2.83	22.86 ± 2.50	0.223
800 m run/s	250.03 ± 23.65	240.33 ± 20.27	0.236
sprint 50/s	9.44 ± 0.77	9.39 ± 0.81	0.874
Sit-ups/pcs	36.90 ± 7.61	34.17 ± 9.19	0.554
Standing long jump/cm	166.55 ± 15.99	168.67 ± 17.98	0.487

Note: “*M*” denotes the mean. “*SD*” denotes standard deviation.

**Table 7:** Pre–post outcomes and mixed-design ANOVA results (*n* = 55).

Outcome	Group	Baseline (*M* ± *SD*)	Post-Intervention (*M* ± *SD*)	*MD*	ANOVA *(F, p,* η^2^*)*											
					Time Effect			Group Effect			Time × Group Effect			Simple Effect Test		
Total Physical Health Score	MRT RT	322.54 ± 24.11 330.31 ± 33.27	376.04 ± 27.69 ** 371.52 ± 32.69 **	53.50 41.21	264.841	0.000 **	0.833	0.047	0.827	0.001	4.456	0.040 *	0.078	193.645 88.973	0.000 ** 0.000 **	0.785 0.627
Waist-to-Hip Ratio	MRT RT	0.76 ± 0.04 0.72 ± 0.03	0.74 ± 0.04 ** 0.72 ± 0.03	−0.02 0.00	9.966	0.003 **	0.158	13.787	0.000 **	0.206	4.305	0.043 *	0.075	15.681 0.519	0.000 ** 0.474	0.228 0.010
Sit-and-reach test	MRT RT	15.71 ± 7.72 17.23 ± 5.46	20.49 ± 6.55 ** 19.19 ± 4.8 **	4.78 1.96	43.945	0.000 **	0.453	0.004	0.948	0.000	7.686	0.008 **	0.127	50.638 6.589	0.000 ** 0.013 *	0.489 0.111
Vital Capacity	MRT RT	2749.32 ± 512.39 2990.79 ± 575.17	3356.84 ± 566.05 ** 3344.79 ± 665.34 **	607.52 354.00	91.579	0.000 **	0.633	0.596	0.444	0.011	6.366	0.015 *	0.107	83.782 22.024	0.000 ** 0.000 **	0.613 0.294
Body fat percentage	MRT RT	25.88 ± 5.99 27.14 ± 5.08	21.30 ± 5.07 ** 23.89 ± 4.44 **	−4.58 −3.25	45.033	0.000 **	0.459	2.223	0.142	0.040	1.299	0.259	0.024	35.309 13.765	0.000 ** 0.000 **	0.400 0.206
800 m run	MRT RT	250.03 ± 23.65 240.33 ± 20.27	236.32 ± 20.52 ** 233.00 ± 22.05 **	−13.71 −7.33	13.628	0.001	0.205	1.583	0.214	0.029	1.251	0.268	0.023	13.256 2.936	0.001 ** 0.092	0.200 0.052
Standing long jump	MRT RT	166.55 ± 15.99 168.67 ± 17.98	174.68 ± 15.19 ** 175.50 ± 20.32 **	8.13 6.83	32.160	0.000	0.378	0.107	0.745	0.002	0.241	0.624	0.005	21.754 11.901	0.000 ** 0.001 **	0.291 0.183
50 m sprint	MRT RT	9.44 ± 0.77 9.39 ± 0.81	9.00 ± 0.52 ** 8.84 ± 0.69 **	−0.44 −0.55	54.515	0.000	0.507	0.379	0.541	0.007	0.574	0.452	0.011	25.150 29.399	0.000 ** 0.000 **	0.322 0.367
Sit-ups	MRT RT	36.90 ± 7.61 34.17 ± 9.19	49.58 ± 9.73 ** 47.54 ± 8.9 **	12.68 13.37	215.086	0.000	0.802	1.134	0.292	0.021	0.154	0.696	0.003	116.716 100.579	0.000 ** 0.000 **	0.686 0.655
Mindfulness	MRT RT	67.00 ± 10.10 71.04 ± 8.24	72.97 ± 9.57 ** 71.50 ± 7.94	5.97 0.46	10.937	0.002 **	0.171	1.346	0.251	0.025	8.039	0.006 **	0.132	21.616 0.099	0.001 ** 0.773	0.290 0.002
Self-Esteem	MRT RT	37.81 ± 7.04 39.33 ± 6.09	40.09 ± 5.62 * 37.31 ± 5.16	2.28 2.02	0.025	0.876	0.195	0.195	0.661	0.004	7.519	0.008 **	0.124	4.817 2.963	0.033 * 0.091	0.083 0.053
Subjective Well-Being	MRT RT	47.23 ± 10.86 47.88 ± 8.63	51.35 ± 9.43 ** 46.38 ± 7.82	4.12 1.50	1.210	0.277	0.022	0.924	0.341	0.017	5.539	0.022 *	0.095	6.830 0.698	0.012 * 0.407	0.114 0.013

Note: (1) * *p* < 0.05, ** *p* < 0.01; (2) MRT refers to the Mindfulness-Enhanced Resistance Training Group, and RT refers to the Resistance Training Group; (3) η^2^: (0.01: small effect, 0.06: moderate effect, 0.14: large effect). A *p*-value less than 0.05 was regarded as statistically significant; (4) *MD* refers to mean difference.

**Table 8:** Decomposition of the total effect into direct and indirect effects.

Regression Equation (*n* = 55)			Fit Indices			Coefficient Significance
Dependent Variable	Predictor Variables	*R*	*R* ^2^	*F*	*β*	*t*
Change in Subjective Well-Being	Group	0.308	0.095	5.539	5.629	2.354 *
Change in Self-Esteem	Group	0.352	0.124	7.519	4.332	2.742 **
Change in Subjective Well-Being	Group	0.659	0.434	19.927	1.614	0.791
	Self-Esteem				0.927	5.582 ***
Change in Subjective Well-Being	Group	0.308	0.095	5.539	5.629	2.354 *
Change in Mindfulness	Group	0.363	0.132	8.039	5.509	2.835 **
Change in Subjective Well-Being	Group	0.347	0.121	3.566	4.480	1.754
	Group				0.209	1.240

Note: “*t*” represents the t-statistic for testing whether each regression coefficient differs significantly from zero; *, **, and *** indicate significance at the 0.05, 0.01, and 0.001 levels, respectively.

**Table 9:** Mediation model testing for changes in self-esteem and mindfulness levels.

Independent Variable	Dependent Variable	Mediator		Effect Size	Bootstrapped	LLCI	ULCI	Effect Size Measure
Group	Change in Subjective Well-Being	Change in Self-Esteem	Total effect	5.629	2.392	0.8316	10.4264	
			Direct effect	1.614	2.040	−2.4801	5.7085	28.68%
			Indirect effect	4.015	1.867	0.9607	8.1125	71.32%
Group	Change in Subjective Well-Being	Change in Mindfulness	Total effect	5.629	2.392	0.8316	10.4264	
			Direct effect	4.480	2.554	−0.6450	9.6047	79.59%
			Indirect effect	1.149	1.535	−1.0895	4.9155	20.41%

Note: LLCI refers to the lower limit of the 95% interval of the estimate; ULCI refers to the upper limit of the 95% interval of the estimate.

## Data Availability

The data supporting this study’s findings are available from the first author (quping@mail.sysu.edu.cn) upon reasonable request.

## Supplementary Materials

The following supporting information can be downloaded at: https://www.mdpi.com/article/10.3390/bs16040553/s1.
