# Supplementary material for: The Influence of Mindfulness-Enhanced Resistance Training Program on the Subjective Well-Being of Female College Students: A Randomized Controlled Trial
Source: Behav Sci (Basel). 2026 Apr 8;16(4):553. doi: 10.3390/bs16040553 (PMC13113385; doi:10.3390/bs16040553)
Supplement: Supplementary file 1 [file behavsci-16-00553-s001.zip › behavsci-4103835-supplementary.pdf]

Contents of Intervention Programs between the Two Groups

**Session**		Mindfulness-Enhanced Resistance Training	Resistance Training
**1**	**Major contents**	1. Introduction to Resistance and Mindfulness<br>2. Training requirements<br>3. Safety education<br>4. The significance of diet in resistance training<br>**5. Mindfulness practice: Mindful Raisin Exercise**	1. Introduction to Resistance<br>2. Training requirements<br>3. Safety education<br>4. The significance of diet in resistance training
**1**	**Independent Tasks**	1. Reading materials: Introduction to mindfulness/fitness diet - carbohydrates<br>2. Daily Fitness and Diet Record<br>3. Mindful eating practice feelings journal	1. Reading materials: Introduction to fitness diet - carbohydrates<br>2. Daily Fitness and Diet Record
**2**	**Major contents**	1. Review & Discussion<br>2. Core Training<br>• Dynamic Abdominal Activations<br>• Crunches<br>• Dynamic Hip Bridges<br>• Dead Bug Exercises<br>• Kneeling Planks<br>• Leg Raises/Hip Raises<br>**3. Mindfulness practice: Awareness of Body Sensations**	1. Review<br>2. Core Training<br>• Dynamic Abdominal Activations<br>• Crunches<br>• Dynamic Hip Bridges<br>• Dead Bug Exercises<br>• Kneeling Planks<br>• Leg Raises/Hip Raises
**2**	**Independent Tasks**	1. Independent fitness<br>2. Reading materials: Introduction to fitness diet - protein<br>3. Focus on a daily activity and record your feelings<br>4. Daily Fitness and Diet Record	1. Independent fitness<br>2. Reading materials: Introduction to Fitness diet - protein<br>3. Daily Fitness and Diet Record

Session		Mindfulness-Enhanced Resistance Training	Resistance Training
**3**	**Major contents**	1. Review & Discussion<br>2. Chest Training<br>• Kneeling/Incline/Narrow Push-Ups<br>• Dumbbell/Barbell Bench Presses<br>• Smith Machine Bench Presses<br>• Cable Chest Flys<br>• Chest Dips<br>• Pec Deck Flys<br>**3. Mindfulness practice: Body Scan**	1. Review<br>2. Chest Training<br>• Kneeling/Incline/Narrow Push-Ups<br>• Dumbbell/Barbell Bench Presses<br>• Smith Machine Bench Presses<br>• Cable Chest Flys<br>• Chest Dips<br>• Pec Deck Flys
**3**	**Independent Tasks**	1. Reading materials:<br>• Observing your own bodily responses<br>• Body scan<br>• Introduction to fitness diet - fat<br>2. Independent fitness<br>3. Mindfulness practice: mindfulness activities in daily life<br>4. Daily Fitness and Diet Record	1. Independent fitness<br>2. Reading materials: Introduction to fitness diet - fat<br>3. Daily Fitness and Diet Record
**4**	**Major contents**	1. Review & Discussion<br>2. Shoulder Training<br>• Shoulder Joint Horizontal Rotations, Resistance Band Shoulder Rotations<br>• Standing Y-W ExercisesSeated<br>• Standing Dumbbell Presses<br>• Dumbbell Lateral Raises<br>• Dumbbell Arnold Presses<br>• Cable Face Pulls<br>• Dumbbell Flys<br>• Resistance Band Lateral Raises<br>• Smith Machine Flys<br>**3. Mindfulness practice: Mindfulness of Pleasant Events**	1. Review<br>2. Shoulder Training<br>• Shoulder Joint Horizontal Rotations, Resistance Band Shoulder Rotations<br>• Standing Y-W ExercisesSeated<br>• Standing Dumbbell Presses<br>• Dumbbell Lateral Raises<br>• Dumbbell Arnold Presses<br>• Cable Face Pulls<br>• Dumbbell Flys<br>• Resistance Band Lateral Raises<br>• Smith Machine Flys
**4**	**Independent Tasks**	1. Reading materials:<br>• Seizing the moment<br>• The story of Nick Vujicic<br>2. Independent fitness<br>3. Mindfulness practice: pleasant events calendar<br>4. Daily Fitness and Diet Record	1. Independent fitness<br>2. Daily Fitness and Diet Record

**Session**		Mindfulness-Enhanced Resistance Training	Resistance Training
**5**	**Major contents**	1. Review & Discussion<br>2. Hip and Leg Training<br>• Hip Bridge with Resistance Band<br>• Prone Hip Extensions<br>• Goblet Squats<br>• Prone Leg Lifts<br>• Lunge Squats<br>• Straight-Leg Deadlifts<br>**3. Mindfulness practice: Loving Kindness Meditation**	1. Review<br>2. Hip and Leg Training<br>• Hip Bridge with Resistance Band<br>• Prone Hip Extensions<br>• Goblet Squats<br>• Prone Leg Lifts<br>• Lunge Squats<br>• Straight-Leg Deadlifts
**5**	**Independent Tasks**	1. Reading materials: Caring for yourself<br>2. Independent fitness<br>3. Mindfulness practice: loving kindness meditation<br>4. Daily Fitness and Diet Record	1. Independent fitness<br>2. Daily Fitness and Diet Record
**6**	**Major contents**	1. Review & Discussion<br>2. Arm Training<br>• Barbell/Dumbbell Curls<br>• Hammer Curls<br>• Close-Grip Push-Ups<br>• Pull-Ups<br>• Cable Triceps Pushdowns<br>• Supine Bench Arm Curls/Extensions<br>• Overhead Arm Curls/Extensions<br>• Neck Arm Curls/Extensions<br>**3. Mindfulness practice: Breathing Space**	1. Review<br>2. Arm Training<br>• Barbell/Dumbbell Curls<br>• Hammer Curls<br>• Close-Grip Push-Ups<br>• Pull-Ups<br>• Cable Triceps Pushdowns<br>• Supine Bench Arm Curls/Extensions<br>• Overhead Arm Curls/Extensions<br>• Neck Arm Curls/Extensions
**6**	**Independent Tasks**	1. Reading materials: Breathing space<br>2. Independent fitness<br>3. Mindfulness practice: unpleasant events calendar<br>4. Daily Fitness and Diet Record	1. Independent fitness<br>2. Daily Fitness and Diet Record

Session		Mindfulness-Enhanced Resistance Training	Resistance Training
**7**	**Major contents**	1. Review & Discussion<br>2. Back Training<br>• Bent-Over W-Y/W-A movements<br>• Bent-Over W-Y/W-A Movements<br>• Bent-Over Rows<br>• Resistance Band High Pulldowns<br>• Pull-Ups<br>• Chin-Ups<br>• T-Bar Rows<br>**3. Mindfulness practice: Living with Stress**	1. Review<br>2. Back Training<br>• Bent-Over W-Y/W-A movements<br>• Bent-Over W-Y/W-A Movements<br>• Bent-Over Rows<br>• Resistance Band High Pulldowns<br>• Pull-Ups<br>• Chin-Ups<br>• T-Bar Rows
**7**	**Independent Tasks**	1. Reading materials: The open mind<br>2. Independent fitness<br>3. Mindfulness practice: breathing space<br>4. Daily Fitness and Diet Record	1. Independent fitness<br>2. Daily Fitness and Diet Record
**8**	**Major contents**	1. Review & Discussion<br>2. Chest Training<br>• Kneeling/Incline/Narrow Push-Ups<br>• Dumbbell/Barbell Bench Presses<br>• Dumbbell Flys<br>• Smith Machine Bench Presses<br>• Pec Deck Flys<br>• Partner Relaxation Stretches<br>**3. Mindfulness practice: Cultivate Gratitude Heart**	1. Review<br>2. Chest Training<br>• Kneeling/Incline/Narrow Push-Ups<br>• Dumbbell/Barbell Bench Presses<br>• Dumbbell Flys<br>• Smith Machine Bench Presses<br>• Pec Deck Flys<br>• Partner Relaxation Stretches
**8**	**Independent Tasks**	1. Reading materials: A grateful heart<br>2. Independent fitness<br>3. Mindfulness practice: loving kindness meditation and grateful event record<br>4. Daily Fitness and Diet Record	1. Independent fitness<br>2. Daily Fitness and Diet Record

**Session**		Mindfulness-Enhanced Resistance Training	Resistance Training
**9**	**Major contents**	1. Introduction to Mindfulness Day<br>2. Review, share, and discuss personal experiences in the program<br>3. Core Training<br>• Dynamic Abdominal Activations<br>• Crunches<br>• Bent-Over Jacks<br>• Russian Twists<br>• Supine Leg Raises<br>**4. Mindfulness practice: Brief Loving Kindness Meditation**	1. Review<br>2. Core Training<br>• Dynamic Abdominal Activations<br>• Crunches<br>• Bent-Over Jacks<br>• Russian Twists<br>• Supine Leg Raises
**9**	**Independent Tasks**	1. Reading materials:<br>The mindfulness journey: bring mindfulness into your life<br>2. Independent fitness<br>3. Mindfulness practice: experience and record mindfulness day<br>4. Daily Fitness and Diet Record	1. Independent fitness<br>2. Daily Fitness and Diet Record
**10**	**Major contents**	1. Review & Discussion<br>2. Gym Free Training<br>• Dynamic Stretching<br>• Review old exercises: chest, shoulders, arms, waist, legs<br>• Free practice<br>**3. Mindfulness practice: Mindful Breathing**	1. Review<br>2. Gym Free Training<br>• Dynamic Stretching<br>• Review old exercises: chest, shoulders, arms, waist, legs<br>• Free practice
**10**	**Independent Tasks**	1. Mindfulness journal prompt: Notice the tastes, colors, smells, and textures of the food when eating<br>2. Mindfulness practice: mindful breathing<br>3. Independent fitness<br>4. Daily Fitness and Diet Record	1. Independent fitness<br>2. Daily Fitness and Diet Record

**Session**		Mindfulness-Enhanced Resistance Training	Resistance Training
**11**	**Major contents**	1. Discussion: mindfulness journal<br>2. Hip and Leg Training<br>• Hip Bridge with Resistance Band<br>• Prone Leg Lifts<br>• High Goblet Squats<br>• Smith Machine Squats<br>• Lunge Squats<br>• Side Lunges<br>• Sumo Deadlifts<br>**3. Mindfulness practice: Body Scan**	1.Hip and Leg Training<br>• Hip Bridge with Resistance Band<br>• Prone Leg Lifts<br>• High Goblet Squats<br>• Smith Machine Squats<br>• Lunge Squats<br>• Side Lunges<br>• Sumo Deadlifts
**11**	**Independent Tasks**	1. Independent fitness<br>2. Mindfulness journal prompt: Pause, and notice the beauty around you.<br>3. Mindfulness practice: body scan<br>4. Daily Fitness and Diet Record	1. Independent fitness<br>2. Daily Fitness and Diet Record
**12**	**Major contents**	1. Review & Discussion<br>2. Arm Training<br>• Hammer Curls<br>• Cable Triceps Pushdowns<br>• Bent-Over Dumbbell Triceps Extensions<br>• Neck Arm Curls/Extensions<br>**3. Mindfulness practice: Breathing Space**	1. Review<br>2. Arm Training<br>• Hammer Curls<br>• Cable Triceps Pushdowns<br>• Bent-Over Dumbbell Triceps Extensions<br>• Neck Arm Curls/Extensions
**12**	**Independent Tasks**	1. Independent fitness<br>2. Mindfulness journal prompt: Pause, and notice the smells around you.<br>3. Mindfulness practice: experience and record mindfulness day<br>4. Daily Fitness and Diet Record	1. Independent fitness<br>2. Daily Fitness and Diet Record

Session		Mindfulness-Enhanced Resistance Training	Resistance Training
**13**	**Major contents**	1. Review & Discussion<br>2. Core Training<br>• Jumping Jacks<br>• High Knees<br>• Plank Jacks<br>• Plank, Side Plank<br>• Hip Raises (Prone)<br>• Hip Thrusts (Supine)<br>**3. Mindfulness practice: Loving Kindness Meditation**	1. Review<br>2. Core Training<br>• Jumping Jacks<br>• High Knees<br>• Plank Jacks<br>• Plank, Side Plank<br>• Hip Raises (Prone)<br>• Hip Thrusts (Supine)
**13**	**Independent Tasks**	1. Independent fitness<br>2. Mindfulness journal prompt: Pause, and notice the sounds around you.<br>3. Mindfulness practice: loving kindness meditation<br>4. Daily Fitness and Diet Record	1. Independent fitness<br>2. Daily Fitness and Diet Record
**14**	**Major contents**	1. Review & Discussion<br>2. Shoulder Training<br>• Resistance Band Pulls, Shoulder Press<br>• Dumbbell Presses<br>• Dumbbell Arnold Presses<br>• Dumbbell Lateral Raises, Flyes<br>• Pike Push-Ups<br>**3. Mindfulness practice: Notice the Pleasant Moments**	1. Review<br>2. Shoulder Training<br>• Resistance Band Pulls, Shoulder Press<br>• Dumbbell Presses<br>• Dumbbell Arnold Presses<br>• Dumbbell Lateral Raises, Flyes<br>• Pike Push-Ups
**14**	**Independent Tasks**	1. Independent fitness<br>2. Mindfulness journal prompt: Pause, and put your mind to one thing.<br>3. Mindfulness practice: experience and record mindfulness day<br>4. Daily Fitness and Diet Record	1. Independent fitness<br>2. Daily Fitness and Diet Record

Session		Mindfulness-Enhanced Resistance Training	Resistance Training
**15**	**Major contents**	1. Review & Discussion<br>2. Back Training<br>• Prone A Extensions, Supermans<br>• Barbell/Dumbbell Rows<br>• Dumbbell Single-Arm Rows<br>• Pull-Ups<br>• Lat Pulldowns<br>• Resistance Band Bent-Over Rows<br>**3. Mindfulness practice: Body Scan**	1. Review<br>2. Back Training<br>• Prone "A" Extensions, Supermans<br>• Barbell/Dumbbell Rows<br>• Dumbbell Single-Arm Rows<br>• Pull-Ups<br>• Lat Pulldowns<br>• Resistance Band Bent-Over Rows
**15**	**Independent Tasks**	1. Independent fitness<br>2. Mindfulness journal prompt: Pause, and pay attention to the things in your life that are about to be evaluated or perceived to be evaluated. Try not to evaluate, and continue to write about your feelings with original insight, experience, acceptance, and understanding.<br>3. Mindfulness practice: mindful breathing; body scan<br>4. Daily Fitness and Diet Record	1. Independent fitness<br>2. Daily Fitness and Diet Record
**16**	**Major contents**	1. Review & Discussion<br>2. Core and Leg Training<br>• Jogging<br>• Hip Band Squats, High Goblet Squats<br>• Neck Squats<br>• Sumo Squats<br>• Bulgarian Squats<br>• Plank<br>• V-Plank<br>**3. Mindfulness practice: Body Scan**	1. Review<br>2. Core and Leg Training<br>• Jogging<br>• Hip Band Squats, High Goblet Squats<br>• Neck Squats<br>• Sumo Squats<br>• Bulgarian Squats<br>• Plank<br>• V-Plank

Session		Mindfulness-Enhanced Resistance Training	Resistance Training
16	**Independent Tasks**	1. Independent fitness<br>2. Mindfulness journal prompt: Pause, try to accept and understand the things or moments in your life that you are resisting or preparing to resist (specifically, things or moments that are objective and cannot be changed for the time being), and write about your feelings.<br>3. Mindfulness practice: experience and record mindfulness day	1. Independent fitness<br>2. Daily Fitness and Diet Record
17	**Major contents**	1. Review & Discussion<br>2. Core and Arm Training<br>• Dumbbell Bicep Curls, Hammer Curls<br>• Barbell Curls<br>• Dumbbell Triceps Extensions<br>• Barbell Triceps Extensions<br>• Plank Jacks<br>• Burpees<br>**3. Mindfulness practice: Loving Kindness Meditation**	1. Review<br>2. Core and Arm Training<br>• Dumbbell Bicep Curls, Hammer Curls<br>• Barbell Curls<br>• Dumbbell Triceps Extensions<br>• Barbell Triceps Extensions<br>• Plank Jacks<br>• Burpees
17	**Independent Tasks**	1. Independent fitness<br>2. Mindfulness journal prompt: Pause, and try to be aware and accepting of the impatient moment when practicing resistance training, or the moment in daily life. Write about your feelings.<br>3. Mindfulness practice: loving kindness meditation<br>4. Daily Fitness and Diet Record	1. Independent fitness<br>2. Daily Fitness and Diet Record

Session		Mindfulness-Enhanced Resistance Training	Resistance Training
**18**	**Major contents**	1. Review & Discussion<br>2. Barbell and Battle Rope Training<br>• Squats, Deadlifts, Rows<br>• Bicep Curls, Hammer Curls, Overhead Triceps Extensions<br>• Shoulder Presses, Chest Flys, Front Raises<br>• Battle Rope: Continuous Fast Waves, Powerful Rope Slams<br>**3. Mindfulness practice: Body Scan**	1. Review<br>2. Barbell and Battle Rope Training<br>• Squats, Deadlifts, Rows<br>• Bicep Curls, Hammer Curls, Overhead Triceps Extensions<br>• Shoulder Presses, Chest Flys, Front Raises<br>• Battle Rope: Continuous Fast Waves, Powerful Rope Slams
**18**	**Independent Tasks**	1. Independent fitness<br>2. Mindfulness journal prompt: Record a moment of trust in yourself or others.<br>3. Mindfulness practice: experience and record mindfulness day<br>4. Daily Fitness and Diet Record	1. Independent fitness<br>2. Daily Fitness and Diet Record
**19**	**Major contents**	1. Review & Discussion<br>2. Pair Training<br>• Chase Running<br>• Partner Push-ups, Rows, Pulls, Lunges<br>• Partner Push-ups, Squats<br>**3. Mindfulness practice: Loving Kindness Meditation**	1. Review<br>2. Pair Training<br>• Chase Running<br>• Partner Push-ups, Rows, Pulls, Lunges<br>• Partner Push-ups, Squats
**19**	**Independent Tasks**	1. Independent fitness<br>2. Mindfulness journal prompt: Write down one or two recent thoughts or moments that you can't let go, try to let it go.<br>3. Mindfulness practice: experience and record mindfulness day<br>4. Daily Fitness and Diet Record	1. Independent fitness<br>2. Daily Fitness and Diet Record

Session		Mindfulness-Enhanced Resistance Training	Resistance Training
**20**	**Major contents**	1. Review & Discussion<br>2. Gym Free Training<br>• Dynamic Stretching<br>• Review Old Exercises: Chest, Shoulders, Arms, Waist, Legs<br>• Free Practice<br>**3. Mindfulness practice: Loving Kindness Meditation; Breathing Space**	1. Review<br>2. Gym Free Training<br>• Dynamic Stretching<br>• Review Old Exercises: Chest, Shoulders, Arms, Waist, Legs<br>• Free Practice
**20**	**Independent Tasks**	1. Independent fitness<br>2. Mindfulness journal prompt: Write about a recent event or moment that you would have been striving or forcing, but are not striving now. How do you feel?<br>3. Mindfulness practice: loving kindness meditation; breathing space<br>4. Daily Fitness and Diet Record	1. Independent fitness<br>2. Daily Fitness and Diet Record
**21**	**Major contents**	1. Review & Discussion<br>2. Gym Free Training<br>• Dynamic Stretching<br>• Review Old Exercises: Chest, Shoulders, Arms, Waist, Legs<br>• Free Practice<br>**3. Mindfulness practice: Notice the Unpleasant Moments and Try to Accept; Breathing Space**	1. Review<br>2. Gym Free Training<br>• Dynamic Stretching<br>• Review Old Exercises: Chest, Shoulders, Arms, Waist, Legs<br>• Free Practice
**21**	**Independent Tasks**	1. Independent fitness<br>2. Mindfulness journal prompt: This week, what is the most impressive or interesting knowledge do you think when you attended a class that usually bored you?<br>3. Mindfulness practice: experience and record mindfulness day	1. Independent fitness<br>2. Daily Fitness and Diet Record

Session		Mindfulness-Enhanced Resistance Training	Resistance Training
**22**	**Major contents**	1. Review & Discussion<br>2. Resistance Band Double Training (Hip and Leg)<br>• Jogging<br>• Resistance Band Partner Exercises<br>• Lunge Squats, Twists, Squats, Scissor Jumps<br>• Clap Jumping Jacks, Alternating Handshakes, Squats<br>**3. Mindfulness practice: Loving Kindness Meditation**	1. Review<br>2. Resistance Band Double Training (Hip and Leg)<br>• Jogging<br>• Resistance Band Partner Exercises<br>• Lunge Squats, Twists, Squats, Scissor Jumps<br>• Clap Jumping Jacks, Alternating Handshakes, Squats
**22**	**Independent Tasks**	1. Independent fitness<br>2. Mindfulness journal prompt: List 1–3 parts of your body that you are most satisfied with.<br>3. Mindfulness practice: loving kindness meditation<br>4. Daily Fitness and Diet Record	1. Independent fitness<br>2. Daily Fitness and Diet Record
**23**	**Major contents**	1. Review & Discussion<br>2. Barbell and Resistance Band Training<br>• Warm-up Jogging<br>• Resistance Band Partner Exercises: Lunge Squats + Squats, Left and Right Lunges + High Knees<br>• Dumbbell Bench Presses<br>• Dumbbell Flyes<br>• Dumbbell Curls/Extensions<br>**3. Mindfulness practice: Breathing Space**	1. Review<br>2. Barbell and Resistance Band Training<br>• Warm-up Jogging<br>• Resistance Band Partner Exercises: Lunge Squats + Squats, Left and Right Lunges + High Knees<br>• Dumbbell Bench Presses<br>• Dumbbell Flyes<br>• Dumbbell Curls/Extensions
**23**	**Independent Tasks**	1. Independent fitness<br>2. Mindfulness journal prompt: Which good habits, although small, have benefited you all the time?<br>3. Mindfulness practice: experience and record mindfulness day<br>4. Daily Fitness and Diet Record	1. Independent fitness<br>2. Daily Fitness and Diet Record

**Session**		Mindfulness-Enhanced Resistance Training	Resistance Training
**24**	**Major contents**	1. Review & Discussion<br>2. Core Training<br>• Partner Plank High-Fives<br>• Partner Push-Up High-Fives<br>• Partner Squats<br>**3. Mindfulness practice: Body Scan**	1. Review<br>2. Core Training<br>• Partner Plank High-Fives<br>• Partner Push-Up High-Fives<br>• Partner Squats
**24**	**Independent Tasks**	1. Independent fitness<br>2. Mindfulness practice: body scan<br>3. Daily Fitness and Diet Record	1. Independent fitness<br>2. Daily Fitness and Diet Record
**25**	**Major contents**	1. Review & Discussion<br>2. Shoulder Training<br>• Resistance Band Pulls, Shoulder Press<br>• Dumbbell Presses<br>• Dumbbell Arnold Presses<br>• Dumbbell Lateral Raises, Flyes<br>• Pike Push-Ups<br>**3. Mindfulness practice: Notice the Pleasant Moments**	1. Review<br>2. Shoulder Training<br>• Resistance Band Pulls, Shoulder Press<br>• Dumbbell Presses<br>• Dumbbell Arnold Presses<br>• Dumbbell Lateral Raises, Flyes<br>• Pike Push-Ups
**25**	**Independent Tasks**	1. Independent fitness<br>2. Mindfulness journal prompt: Pause, and record the two most impressive knowledge points from this week's class.<br>3. Mindfulness practice: experience and record mindfulness day<br>4. Daily Fitness and Diet Record	1. Independent fitness<br>2. Daily Fitness and Diet Record
**26**	**Major contents**	1. Review & Discussion<br>2. Chest Training<br>• Kneeling/Incline/Narrow Push-ups<br>• Dumbbell/Barbell Bench Presses<br>• Smith Machine Bench Presses<br>• Pec Deck Flys<br>• Partner Relaxation Stretches<br>**3. Mindfulness practice: Loving Kindness Meditation**	1. Review<br>2. Chest Training<br>• Kneeling/Incline/Narrow Push-ups<br>• Dumbbell/Barbell Bench Presses<br>• Smith Machine Bench Presses<br>• Pec Deck Flys<br>• Partner Relaxation Stretches

Session		Mindfulness-Enhanced Resistance Training	Resistance Training
26	**Independent Tasks**	1. Independent fitness<br>2. Mindfulness practice: loving kindness meditation<br>3. Daily Fitness and Diet Record	1. Independent fitness<br>2. Daily Fitness and Diet Record
27	**Major contents**	1. Review & Discussion<br>2. Arm Training<br>• Dumbbell Bicep Curls, Hammer Curls<br>• Barbell Curls<br>• Dumbbell Triceps Extensions<br>• Barbell Triceps Extensions<br>**3. Mindfulness practice: Cultivate Gratitude Heart**	1. Review<br>2. Arm Training<br>• Dumbbell Bicep Curls, Hammer Curls<br>• Barbell Curls<br>• Dumbbell Triceps Extensions<br>• Barbell Triceps Extensions
27	**Independent Tasks**	1. Independent fitness<br>2. Mindfulness journal prompt: Pause, and record one person you felt grateful to this week.<br>3. Mindfulness practice: breathing space<br>4. Daily Fitness and Diet Record	1. Independent fitness<br>2. Daily Fitness and Diet Record
28	**Major contents**	1. Review & Discussion<br>2. Hip and Leg Training<br>• Hip Bridge, Resistance Band<br>• Prone Hip Extensions<br>• Prone Leg Lifts<br>• High Goblet Squats<br>• Smith Machine Squats<br>• Lunge Squats<br>• Deadlifts<br>**3. Mindfulness practice: Body Scan**	1. Review<br>2.Hip and Leg Training<br>• Hip Bridge, Resistance Band<br>• Prone Hip Extensions<br>• Prone Leg Lifts<br>• High Goblet Squats<br>• Smith Machine Squats<br>• Lunge Squats<br>• Deadlifts
28	**Independent Tasks**	1. Independent fitness<br>2. Mindfulness journal prompt: Pause, and feel the moment or movement with the strongest muscle sensation during resistance training.<br>3. Mindfulness practice: body scan<br>4. Daily Fitness and Diet Record	1. Independent fitness<br>2. Daily Fitness and Diet Record

Session		Mindfulness-Enhanced Resistance Training	Resistance Training
**29**	**Major contents**	1. Review & Discussion<br>2. Back Training<br>• Bent-Over W-Y/W-A Movements<br>• Bent-Over Rows<br>• Resistance Band, High Pulldowns<br>• Pull-Ups<br>• T-Bar Rows<br>**3. Mindfulness practice: Living with Stress**	1. Review<br>2. Back Training<br>• Bent-Over W-Y/W-A Movements<br>• Bent-Over Rows<br>• Resistance Band, High Pulldowns<br>• Pull-Ups<br>• T-Bar Rows
**29**	**Independent Tasks**	1. Independent fitness<br>2. Mindfulness journal prompt: Pause, and record the two most stressful moments this week.<br>3. Mindfulness practice: breathing space<br>4. Daily Fitness and Diet Record	1. Independent fitness<br>2. Daily Fitness and Diet Record
**30**	**Major contents**	1. Review & Discussion<br>2. Core Training<br>• Partner Plank High-Fives<br>• Partner Push-Up High-Fives<br>• Partner Squats<br>3. Review, share and discuss personal experiences from the course	1. Review<br>2. Core Training<br>• Partner Plank High-Fives<br>• Partner Push-Up High-Fives<br>• Partner Squats<br>3. Review, share and discuss personal experiences from the course
**30**	**Independent Tasks**	/	/
